# Influence of sub-national social health insurance scheme on enrollees’ health seeking behaviour in Anambra state, Nigeria: a pre and post study

**DOI:** 10.1186/s12889-022-13606-x

**Published:** 2022-06-11

**Authors:** Simeon Onyemaechi, Uchenna Ezenwaka

**Affiliations:** 1 Anambra State Health Insurance Agency (ASHIA), Awka, Anambra State Nigeria; 2 Department of Health Administration and Management, Faculty of Health Science and Technology, College of Medicine, University of Nigeria Enugu Campus, Enugu, Nigeria; 3 Health Policy Research Group, College of Medicine, University of Nigeria Enugu Campus, Enugu, Nigeria

**Keywords:** Enrollees, Health insurance, Health seeking behaviour, Healthcare utilization, Nigeria, Social state health insurance scheme

## Abstract

**Background:**

Appropriate health-seeking behaviour (HSB) is crucial for improving health outcomes and achieving universal health coverage (UHC). Accessing healthcare through the state social health insurance scheme (SSHIS) could lead to improved HSB. The study explores the influence of access to healthcare through health insurance on the HSB of the enrollees of the SSHIS in southeast, Nigeria.

**Methods:**

A descriptive cross-sectional study undertaken in twelve health facilities in Anambra state using quantitative and qualitative research methods. Data were collected through a facility-based survey (*n* = 447) and sex-disaggregated focus group discussions (*n* = 12) of health insurance enrollees. Univariate and bivariate analyses were performed for quantitative data, while qualitative data were analyzed using a manual content approach.

**Result:**

The findings revealed a positive change in enrollee’s HSB post-health insurance enrollment. Majority (83%) of the respondents reported that they immediately take action when ill post-health insurance enrollment as against 34% (pre-health insurance) resulting in a 49% increase, with a statistically significant difference (*p* < 0.02). There was a statistically significant association between positive HSB and marital status (*p* < 0.04); educational level (*p* < 0.00); occupation (*p* < 0.03) and ownership of health facility (*p* < 0.00). There was an increase in the percentage of enrollees who use the hospital as their first choice of provider during an illness episode post-health insurance enrollment. This increased from 37.4% to 90.2% (post-health insurance enrollment), representing a 52.8% increase, which is statistically significant (*p* < 0.03), in seeking care in hospitals. Similarly, the percentage (46%) of enrollees using patent medicine vendors (PMVs) as their first choice of provider when ill prior to enrollment in health insurance decreased to 8.1% post-health insurance enrollment, representing a 38% decline with a statistically significant (*p* < 0.00) drop in PMV patronage. Reasons for positive HSB include low cost of services and availability of quality care such as quality drugs, presence of doctors, and other skilled health workers by the health insurance facilities.

**Conclusion:**

Health insurance has been established as an effective strategy for improving appropriate HSB. Hence, increasing coverage of health insurance among the uninsured is crucial in improving access to quality and affordable health care towards achieving UHC, particularly in developing countries.

## Background

Health insurance has been recognized as an important strategy for improving health outcomes, particularly in this era of achieving the Sustainable Development Goal (SDG) of promoting well-being for all [1]. Evidence shows that health insurance reduces the cost of healthcare services including medication, laboratory services, hospitalization etc. thereby enabling improved access to care during illness episode [2, 3]. However, inappropriate or poor health seeking behavoiur (HSB) had been associated with poor health outcomes, higher mortality and increased morbidity [4, 5]. In low-and middle-income countries (LMICs), evidence shows that there is predominantly poor HSB [5] which contributes significantly to a deteriorating health condition due to delays in seeking healthcare resulting to poor treatment outcomes among patients [6, 7].

HSB is referred to as peoples’ action or inaction, or delay undertaken by individuals, following recognition of ill-health for the purpose of finding a suitable remedy to reinstate health [4]. HSB encompasses actions undertaken for the purpose of preventing illness, maintaining good health condition, as well as dealing with departures from a good state of health [8]. It is important to note that the choice of healthcare provider made by an individual or household when responding to perceived ill health or an illness episode is an essential of HSB. Previous studies reveal that several predisposing factors including individual needs, health system factors and treatment options are known to influence people’s HSB which usually culminate in their practice of self-healthcare, timing of seeking care, alternative choice of treatment source etc. before finally opting in for health facility-based healthcare [9].

Several factors contribute or have been linked to poor HSB. For instance, evidence shows that poor HSB leads to low utilization of healthcare and is associated with low incomes, and prohibitive cost of healthcare service [10–12], maldistribution of health facilities in rural and urban areas [10, 11]. However, enrollment in social health insurance scheme (SHIS) could be a way of improving uptake and access to care during illness episode by reducing geographical and financial barriers to healthcare including out-of-pocket expenditure (OOPE).

Globally, social health insurance is recognized as a crucial instrument within the context of health financing reforms towards achieving universal health coverage (UHC) [13]. This is due to its unique characteristics of prepayment method of operation. More so, health insurance as a financing mechanism promotes cross-subsidization of funds, allowing for risk pooling within a health system [14, 15]. Evidence shows that the availability of SHIS significantly improves access and quality of care [16, 17] and removes financial barriers imposed by OOPE for healthcare [17] while lack of it contributes significantly to poor health status among un-insured people [16] and could lead to poor HSB. Hence, the manner a SHIS system is arranged is likely to have influence on HSB and utilization of health services [18, 19].

The Nigerian National Health Insurance Scheme (NHIS) was introduced in 2005, however, it has struggled over the years with only about 5percent population coverage to date [20]. Consequently, the decentralization of the NHIS whereby state governments established respective social health insurance systems such as the Anambra State Health Insurance Scheme (ASHIS)—established in 2016 with the sole aim to address the existing health inequities and high out-of-pocket expenditures (OOPE) to individuals living in the state towards achieving UHC by the year 2030 [21, 22]. It is important to note that the main health financing mechanism in the State is OOPE at 91.1% thus, subjecting many households to the effects of financial catastrophe [23, 24]. Consequently, significantly limits access to healthcare for most of the population. The ASHIS enrollees are entitled to a benefit package which comprise of preventive, promotive and curative services, provided at primary, secondary, and tertiary healthcare facilities taking into cognizance the prevailing local disease burden and morbidity in state. The basic characteristics of the scheme is described in Table 1.

**Table 1:** Overview of the key features of the Anambra State Health Insurance Scheme

Feature	Description
Year of establishment	ASHIA is established by the Anambra state health insurance scheme law in 2016 Officially launched and started its operation in September 2018
Scheme design and management	Operates a single pool system Premium contribution rate is determined by actuary where the contribution is as follows: ◦ Equity fund established for the vulnerable persons and ◦ Earnings-related to the public (State & LGA) and organized private sector (OPS) employees For the public sector, an employer pays 10% while employee contributes 5% of the basic salary. However, for employees of the organized private sector, the employer may decide to pay the entire contribution for the employees Contribution of an annual premium of twelve thousand Naira (N12,000) (24USD) per person, for individuals who are not in a formal employment Co-payment of only 10% of the cost of medications prescribed to an enrollee whether as outpatient or inpatient services made to the health care providers (HCPs) at the point of care Anambra State Health Insurance Agency (ASHIA) regulates/manages the scheme and acts as sole purchasers
Health insurance model	Prepayment system of healthcare financing
Enrolment	Premium cover healthcare benefits for the employee, a spouse and four (4) children below the age of 21 years for formal sector employees However, children above 21 years will be covered through the OPS or private insurance
Eligibility	All Anambra indigenes are eligible to enroll in the scheme
Source of funds	Financed through premium (social security, payroll taxes, and private contributions); government subsidy (general, earmarked taxes, and non-tax revenues), and other sources (donations/philanthropy, donor funds, Basic Health Care Provision Fund)
Benefit package	Covers basic package of services including health promotion, disease prevention, curative and rehabilitative health care services provided at the primary and secondary levels of care
Provider payment mechanism (PPM)	Capitation and fee-for-service (FFS) model Capitation is for primary healthcare while FFS is for secondary and tertiary healthcare
Purchasing	Done by ASHIA
Selective contracting	Services are provided by accredited private and public health facilities or HCPs across the state
Community involvement in scheme design and management	Feedback given at monthly and quarterly review meetings (comprising clients/enrollees, HCPS and ASHIA staff) and is used to improve the scheme design and service delivery

Source: Authors’ compilation from document review (2022)

Studies that investigated the influence of HSB and healthcare utilization in the context of health insurance scheme in Nigeria is limited. Understanding people’s patterns and driving forces behind patients’ behaviors when ill will help government, policymakers, and health service providers in decision making for provision of health services and on the effectiveness of such programs. This study contributes to the discussion on the role of health insurance in improving universal access to healthcare and towards achieving SDGs Goal 3. Specifically, it highlights insights into the question of whether there is an effect between access to health insurance and care seeking behavoiur of the enrollees of the ASHIS in southeast, Nigeria.

## Methods

### Study design and study area

This was a descriptive cross-sectional mixed-method study comprising of quantitative and qualitative data collection methods employing pre- and post-intervention approach. The study was conducted in twelve (12) secondary health facilities selected from six (6) local government areas (LGAs) in Anambra State, located in the southeast region of Nigeria. The LGAs were purposively selected to include the geographic location (rural and urban) and geopolitical spread (three senatorial districts in the state) and type of health facility (private and public).

Anambra state has an estimated annual growth rate of 2.8%, with a projected population of 4.5 million people in 2018 [25]. Anambra is legislatively divided into 3 senatorial districts- South, Central, and North. The State is also divided into 21 local government areas (LGAs) for administrative purposes. Structurally, the State health system is organized into three tiers: primary, secondary, and tertiary level of healthcare. The State Ministry of Health (SMOH) has the mandate of overall coordination of the health system. Healthcare services are provided by both private and public health facilities.

### Study population, sampling and sample size

The study population consisted of all enrollees of the Anambra State Health Insurance Scheme accessing health care in selected health facilities. Non-insured people who also access services at the selected facilities at the time of the survey were excluded from being interviewed. The study also excluded eligible enrollees who refused to give consent to participate in the study.

A multistage sampling method was adopted in this study. At stage one, we stratified the state into three senatorial districts. For stage two, the simple random sampling (SRS) method was used to select six (6) LGAs (two (2) per senatorial district) comprising one urban and one rural LGA to ensure representativeness. In the third stage, we purposively selected twelve (12) facilities (using a list of ASHIA accredited health care providers [HCPs]) from the six LGAs using the following criteria: i) facility location (one urban and one rural); ii) facility ownership (one public and one private) and, iii) the total number of enrollees in a facility (those with above 500 enrollees) in order to ensure that a minimum sample size per facility will be obtained. These were done to enable subgroup analysis of data. In all, a total of 12 health facilities were included in the study. At the final stage, the respondents to the quantitative (survey) and qualitative (focus group discussion [FGD]) were selected purposively based on those who were accessing healthcare services at the time of the data collection at each facility. The number of enrollees interviewed differed remarkedly across the selected facilities.

The Yaro Yamane formula for determining sample size was used. The formula is stated thus: *n* = N/ 1 + N (e)^2^. Where n is the sample size; N is the total population of enrollees accessing care; e is the allowable error of five percent (0.05), and 1 is the constant. A minimum sample size of 402 was estimated and was further increased by 5% for robustness and to account for incomplete responses and or errors in questionnaires.

For the qualitative method, a subset of the surveyed respondent was purposively selected for the FGD in each health facility based on willingness to participate. Twelve (12) FGDs (6 male and 6 female groups) with the enrollees were conducted. Each lasted for 35–40 min and was held in health facilities. Although we planned to have a discussion group of about 8–10 enrollees from each study facility but eventually had 5 to 7 enrollees due to refusal to spend more time in the facilities outside the medical visit considering the health condition as well as delays in scheduling interview appointments within the data collection period.

### Research variables

In this study, we contextualized an appropriate HSB (positive) as seeking healthcare at conventional health facilities such as primary health centers (PHCs), hospitals/clinic including private and public within 24 h of illness episodes or any condition that requires a medical attention. On the other hand, inappropriate HSB (negative) means seeking healthcare from patent medicine vendors (PMVs), self-medication, herbalist/traditional healers, friends/family members or taking no action when ill [26].

The variables of interest in the quantitative study were i) socio-demographic characteristics such as age, gender, location of the facility, ownership of the facility, highest educational level, marital status, and occupation ii) positive change in HSB, and iii) choice of the provider before and during enrollment into SSHIS. For the qualitative study, variables of interest include the first action taken when feeling ill/sick, choice of HCP, and change in HSB, and reason for positive change in HSB among enrollees before and during enrollment into SSHIS.

### Data collection

A pre-tested structured questionnaire developed by the researchers was used to collect information from the enrollees between January and February 2022. Data were collected on the last services accessed at the study facility under the SSHIS. The questions focused on HSB before and during enrollment into SSHIS, the first action taken when sick, choice of HCP, and change in HSB and reason for this. Twelve research assistants were recruited and trained for 3 days to assist with administering the questionnaire. Electronic copies of the questionnaire uploaded to android tablets using the KoBoCollect App were used to collect data over a period of eight days.

The FGD guide was developed by the researchers and validated by a qualitative research expert. Prior to the commencement of the study, the ASHIA HCPs were informed about the study. The FGDs were conducted by trained and experienced qualitative researchers (one moderator and one notetaker) over a period of 12 days. Each interview was audio-recorded, and handwritten notes were also taken.

The quality of data collection was ensured at different steps of the process including training of research assistants, pretesting and revision of tools, supervision of data collection exercise, and digitization/entry into software to limit manual data entry error. Other mechanism for quality assurance used was multiple researchers working on the same data (e.g., coding by at least two researchers). To ensure the trustworthiness of the study, we analyzed each data set from the quantitative and qualitative components separately and triangulated the findings at interpretive level to offset the weakness of each data collection method and enrich the findings from both sources. The tools were validated by expert researchers to ensure internal consistency and transferability.

### Data analysis

The analysis draws from Andersen’s behavioral model of health care use [27] to examine how health insurance affects enrollees' HSB. The model postulates that utilization of health care is influenced by several factors including predisposing factors, enabling factors, and the need to use health services [27, 28].

Quantitative: A total of 447 questionnaires were completely filled. Descriptive analysis was performed using SPSS version 25 software. Positive changes for HSB were disaggregated by respondents’ characteristics as indicated above in the research variable of interest to highlight distribution as well as to test for associations. Frequencies, percentages, and chi-square (Pearson) and *p*-values were performed and presented in Tables.

Qualitative: The audio files were transcribed verbatim in the language of the discussants and were then translated to English. All transcripts were processed and edited using Microsoft Word. Codes were developed and used for the anonymization of each transcript. Data were analyzed using a manual content approach. Initially, all the transcripts were read to familiarize and get a general understanding of the data. Afterward, the richest transcripts were selected for detailed study and for coding. Key themes and sub-themes relating to HSB were generated, forming the initial coding framework. The generated framework was tested on two more transcripts and was refined into a final coding framework. This was then applied to all the transcripts. Four major themes that emerged from transcripts/coding were:1) change in HSB before and during enrollment into the SHIS; 2) the first choice of HCP when sick; 3) reason for positive change in HSB and, 4) experience of service utilization within SSHIS.

## Results

The findings are presented according to the four major themes described above that emerged from the analysis of data.

### Socio-demographic characteristics

The quantitative data shows that the majority (99.8%) of the respondents were Christians and the average number of months of accessing care in their respective health facilities under ASHIS was 10 months. Among the respondents, 62% registered and are accessing services in private facilities while the rest use public facilities. With respect to location of the health facility, urban respondents constitute 76% of them while rural constitutes 23.9% Other details of the respondents are detailed in Table 2.

**Table 2:** Characteristics of the survey and FGD participants

Variables	Survey respondents (*N* = 447)	FGD participants (*N* = 62)
	**n (%)**	**n (%)**
**Age (**M(SD))	42 (13.5)	
20–30	88(19.6)	6(9.7)
31–40	140(31.3)	18(29.0)
41–50	96(21.6)	17(27.4)
51–60	85(19.0)	11(17.7)
61–70	30(6.6)	8(12.9)
71 & above	8(1.6)	2(3.2)
**Sex**		
Male	127(28.4)	23(37.1)
Female	320(71.6)	39(62.9)
**Marital status**		
Married	358(80.1)	52(83.9)
Single	72(16.1)	6(9.7)
Widow/Widowed	17(3.8)	4(6.5)
**Highest educational level**		
Primary	19(4.3)	2(3.2)
Secondary	121(27.1)	27(43.5)
Tertiary	281(62.9)	30(48.4)
Postgraduate	19(4.3)	1(1.6)
Other (Catering school, OND)	6(1.3)	0(0.0)
**Occupation**		
Unemployed	52(11.6)	9(14.5)
Petty Trader	22(4.9)	10(16.1)
Subsistence Farmer	6(1.3)	0(0.0)
Artisan	16(3.6)	12(19.4)
Government Worker	281(62.9)	20(32.3)
Businessperson	31(6.9)	4(6.5)
Employed in private sector	33(7.4)	5(8.1)
Others (retired, pensioner)	6(1.3)	2(3.2)
**Type of illness for which services was accessed in the last visit**		
Arthritis/rheumatism	17(3.8)	10(16.1)
Ear, Nose and Throat problem	28(6.3)	1(1.61)
Hypertension	29(6.5)	7(11.3)
Malaria	183(40.9)	24(38.7)
Maternal and child health services	53(11.9)	15(24.2)
Ulcer Disease	14(3.1)	5(8.01)
Pneumonia	8(1.8)	0(0)
Typhoid	51(11.4)	0(0)
Others (diabetics, infection, diarrhea etc.)	58(14.3)	0(0)
**Total**	**447**	**62**

*N* = denominator (total number of people that responded to the question); *n* = frequency (total number of observations for each outcome)

### Change in HSB pre and during enrollment in SSHIS

**Fig. 1:**
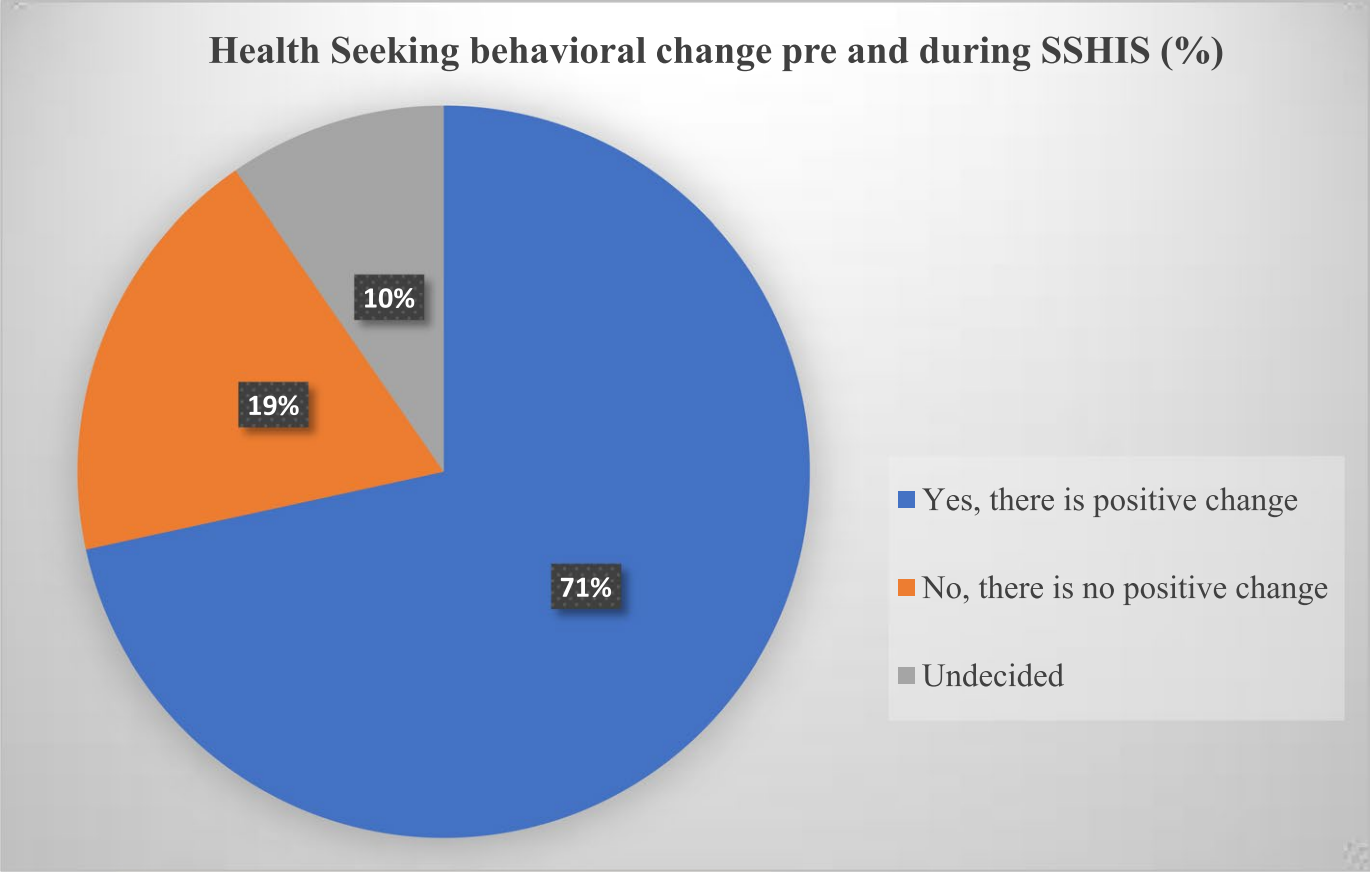
Change in HSB when ill since enrollment and commencement of accessing services under the SSHIS

The analysis of the quantitative study shows that 71% of the respondents affirmed that there is a positive change in their HSB since they enrolled in SSIHS (Fig. 1). To further buttress this, the qualitative study also shows that respondents affirm a positive change in their HSB since they enrolled in the SSHIS. This was evident in the way/action taken in response to ill health or when feeling sick- most of them reported to take immediate action as soon as they observed an ill-health unlike before their enrollment in SSHIS. According to a male respondent, “sometimes, in the past I don't usually come to the hospital immediately I fall sick because of money but now I can access the hospital at will” (Male, Public, Urban—ONONIF01). Another said that enrollment in health insurance, “made him rush to the hospital to save his life unlike before because of the fear of bills from the doctors [laughs]” (Male, Private, Rural- AEAGUF02); “Yes, now anytime I am sick I would just rush to the hospital without wasting any time” (Female, Private, Urban—ASAWKF02).

However, 19% of them reported that their HSB had remained the same even before their enrollment in SSHIS in the state. According to a respondent, “I still behave the same way as before, I will first self-medicate but if I didn't get better, I will go to the hospital” (Female, Public, Rural- IHOKJF01).

There was a statistically significant difference in the percentage of enrollee’s action when sick or feeling sick after their enrollment in SSHIS (Table 3). Majority (83%) of the respondents reported that they immediately take action when sick against 34% resulting in a 49 percent increase. The percentage of those who take action after 24 h to when critically ill before and after enrollment in health insurance decreased too. However, some stated that “It might take 1 week to take action depending on the type of the sickness. It might take 1 week, 2 days, 3 days if the sickness is serious then I will go to the hospital, if it is not serious, I will go to the chemist and take drugs” (Female, Private, Urban—ONONIF02).

**Table 3:** Difference in change among those who reported that there is positive change between before and during enrolment into SSHIS

Behavoiur/Action (*N* = 320)	Before n (%)	During n (%)	Diff n (%)	X^2^ (*p*-value)	Interpretation
Take action immediately I feel sick	109(34.1)	268(83.8)	159(49.7)	4.03 (0.02) *	Increased
Take action within 24 h	72(22.5)	42(13.1)	-30(-9.4)	2.55 (0.03) *	Decreased
Take action 2 days after	62(19.4)	8(2.5)	-54(-16.9)	4.55 (0.03) *	Decreased
Take action one week after	53(16.6)	1(0.3)	-52(-16.3)	1.72 (0.01) *	Decreased
Take action when critically ill	24(7.5)	1(0.3)	-23(-7.2)	2.22 (0.02) *	Decreased

*p*-value < 0.05; * statistically significant value

Table 4 highlights the results of bivariate analysis of demographic factors associated with enrollees HSB. Statistically significant associations were found, i) when ‘positive HSB’ was cross tabulated with ‘enrollees’ marital status' (*p* < 0.04); ii) ‘educational level’ (*p* < 0.02); iii) ‘occupation’ (*p* < 0.03) and ‘ownership of health facility’ (*p* < 0.00).

**Table 4:** Relationship between characteristics of enrollees and positive HSB after enrollment in SSHIS

Characteristic (*N* = 320)	n (%)	X^2^ (*p*-value)
**Geographical location**		
Rural	81(18.1)	1.45(0.49)
Urban	239(53.5)	
**Facility ownership**		
Private	225(50.3)	12.6(0.00) *
Public	95(21.3)	
**Sex**		
Male	97(21.7)	2.23(0.33)
Female	223(49.9)	
**Marital status**		
Married	250(55.9)	9.39(0.04) *
Single	57(12.8)	
Widow/Widowed	13(2.9)	
**Highest educational level**		
Primary	14(3.1)	20.9(0.02) *
Secondary	97(21.7)	
Tertiary	188(42.1)	
Postgraduate	16(3.6)	
Other (Catering school, OND)	5(1.1)	
**Occupation**		
Unemployed	39(8.7)	24.7(0.03) *
Petty Trader	14(3.1)	
Subsistence Farmer	6(1.3)	
Artisan	10(2.2)	
Government Worker	193(43.2)	
Businessperson	26(2.8)	
Employed in private sector	29(6.5)	
Others (retired, pensioner)	3(0.7)	

*p*-value < 0.05; * statistically significant value

### Choice of HCP when sick or feeling sick before and during enrolment into SSHIS

The quantitative analysis shows that there was a statistically significant difference in the percentage of enrollees who started seeking healthcare in health care facilities after their enrollment in SSHIS (Table 5). Some (46%) of the respondents prefer PMVs for treatment when ill but the situation has changed since they enrolled in SSHIS. However, the percentage of those who prefer PMVs reduced to 8.1%, representing a 38% decline among those who patronize them. Although some (37%) respondents claimed that they use hospital before and during enrollment in SSHIS, there is an increase in the number of those who use the hospital as their first point of call when they or any of their household members fall sick resulting in the percentage increase of hospital users by 52%.

**Table 5:** First choice of health care provider when ill/feeling sick before and during enrollment into SSHIS among respondents

Provider type (*N* = 447)	Before n (%)	After n (%)	Diff n (%)	X^2^ (*p*-value)	Interpretation
Patent Medicine Vendor	207 (46.3)	36 (8.1)	-171 (-38.2)	6.03 (0.00) *	Decreased
Herbal traditional healer	7 (1.6)	1(0.2)	-6 (-1.4))	3.55 (0.06)	Decreased
Hospital (Private/public)	167 (37.4)	403 (90.2)	236 (52.8)	4.55 (0.03) *	Increased
Primary health center (PHC)	18 (4.0)	3 (0.7)	-15 (-3.3)	1.72 (0.07)	Decreased
Self- treatment (sought help from family member/friend, self-prescribed drugs)	42 (9.4)	0 (0.0)	-42 (-9.4)	5.99 (0.02) *	Decreased
No Action	6 (1.3)	4 (0.9)	-2 (-0.4)	1.03 (0.12) *	Decreased

Note:—= decline in client patronizing a particular HCP; *p*-value < 0.05; * statistically significant value

Similarly, the qualitative reveals that respondents visit PMVs, self-medication, and herbal medicines as their first choice of HCPs when seeking healthcare -for treatment or purchase medicine when they feel sick or become ill before they enrolled in ASHIA. This according to them has changed as they reported that now that they have been enrolled in the scheme, they access hospitals once they are sick and no longer seek care where they used to attend. In the word of a respondent*,* “when I fall sick, I go to the chemist to buy drugs and drink but since I started using ASHIA, I come to the hospital to receive treatment. (Female, Private, Rural—NJIENUF02). Another respondent said, “since I have been in ASHIA, I have not visited chemist shops, I have been coming here to receive my drugs in this hospital with my insurance card” (Female, Private, Urban—ONONIF02). A respondent who takes herbal concoction said, “when I am sick, I normally take local medicine and take, because I don’t have money, but the world changed now, so I go to the hospital where I am registered with ASHIA and receive treatment and my health much better now” (Male, Private, Rural- AEAGUF02).

However, some respondents claimed that hospitals have remained their first point of seeking healthcare when sick even before they joined ASHIA. “Whenever I am sick or my children are sick, we go to hospital and has remained so before and after joining the health insurance scheme” (Female, Private, Urban- ASAWKF02).

### Reason for positive change in HSB among the enrollees

**Fig. 2:**
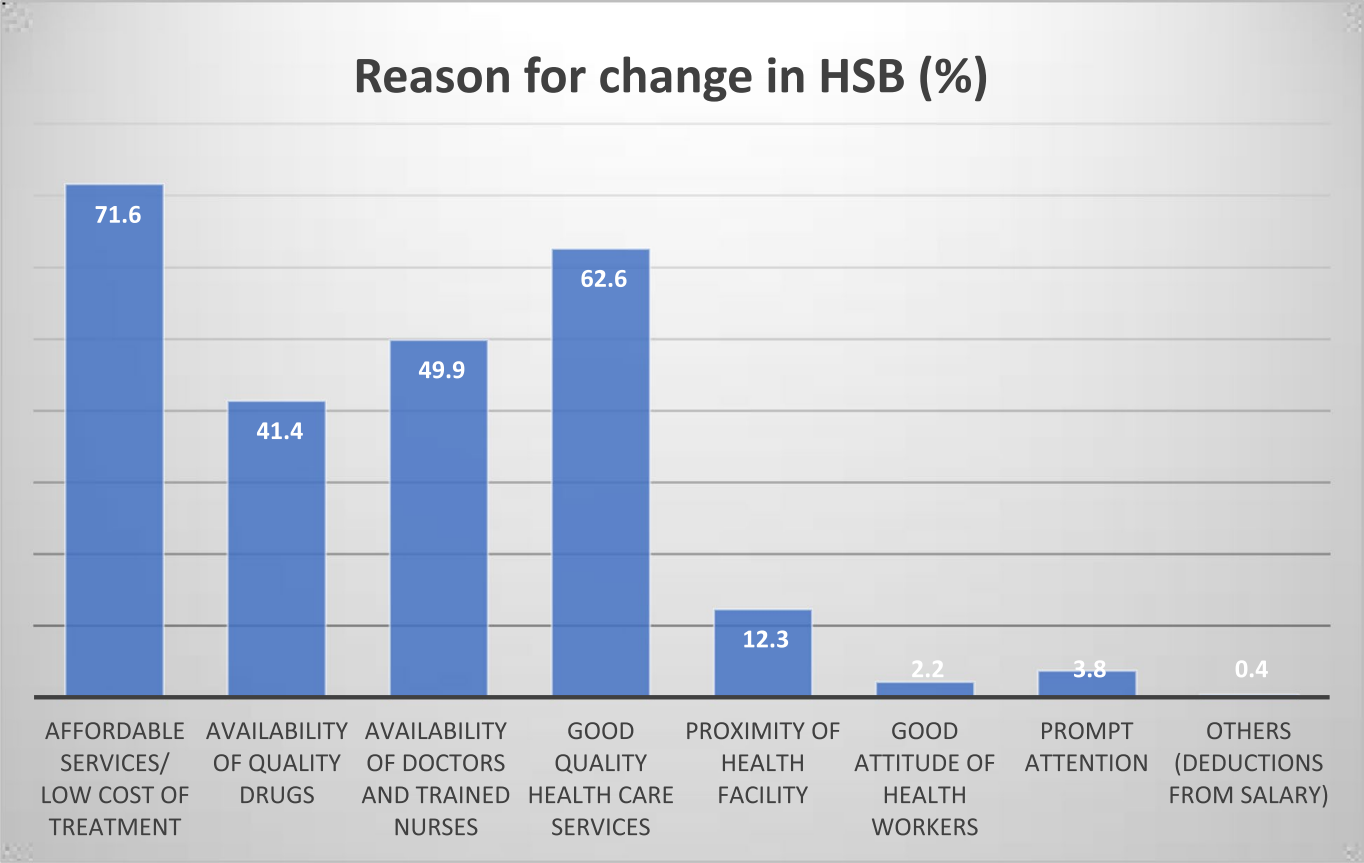
Main reasons for change in HSB (multiple choose response option)

Figure 2 highlights the reason for the HSB observed among the respondents. Majority (71.6%) of them stated that the non-payment/low cost of services they receive is the reason for the positive change in their HSB. Other reasons are better quality of care (62.6%), availability of trained health providers and drugs—49.9% and 41.4% respectively.

According to the FGD participants, the positive change in their HSB after registering and commencing utilization of healthcare services is because of the following reasons: i) Affordable healthcare services/low cost of services at the point of payment. A respondent said, “since I joined ASHIA, they reduced my health spending since I started coming here (ASHIA HCP) they have not charged me even 1 Naira. After treating me I go home” (Female, Public, Rural—NJIENUF01). Another said, “since I registered for this health insurance, I get treatment and even collect drugs even though I don't have any money” (Female, Public, Urban—ASAWKF01), “In the past I normally spent a lot of money forcing me to always procure drugs from the chemist instead of visiting the hospital. But when I joined ASHIA, it is less, so no need buying drugs from the chemist or visiting the quack health providers, ASHIA treats people very well, and will look after you very well” (Male, Private, Rural- AEAGUF02) and; ii) Presence and availability of doctors and qualified health workers – “the quality of the services we get from ASHIA, especially from the doctors is beautiful. (Male, Public, Urban—ONONIF01); iii) Friendly/good attitude of health workers during treatment or check-up; iv) Quality of services offered by the HCPs – “Yes, there’s a difference, before I always suffer from malarial and typhoid but now, I have entered ASHIA, there’s a huge difference. ….. Since I entered ASHIA and received treatment, malarial and typhoid were completely cured (Female, Public, Rural—AEUMUF01). To further buttress this, respondents stated,

“In the chemist where I used to buy drugs … But they didn’t give me treatment to my satisfaction … under ASHIA I registered, I now change it to the general hospital, since I started coming here (The general Hospital) there are huge differences, at least when you come here, they will check your weight, BP and everything you desire. You will see the doctor but, in that place, (Chemist or Health center) there’s no doctor but here you will see a doctor and explain everything that’s wrong with you and the doctor will prescribe drugs for you and you will get better after taking the drugs (Female, Public, Rural—AEUMUF01).

“To tell you the truth since I started coming here, I registered under ASHIA and started coming here for treatment, the way they look after me is exceptional because once it’s your turn, you will see a doctor and explain your ailment to him, the drugs he prescribed for you once you finished it like I always try and finish any drugs prescribed for me. Once you finished the drugs with the injections, my ailment will stop. Another thing is that the way the staff of this hospital takes care of the people is very good both the senior and junior staff take care of people very well and that made me always visit here instead of going to the chemist” (Female, Public, Rural—AEUMUF01).

However, a respondent said that the reason for their change in their HSB is because of health insurance contribution deductions from the salaries of civil servants, although her experience accessing healthcare in ASHIA HCP has been an improvement in her health. In the word of a civil servant, “they told us that are civil servants that whether you register or not they will deduct 2000 naira from your salary, so I don’t have option than to register and it's better for me in terms of my health” (Female, Private, Rural—NJIENUF02).

These reasons were reported by the respondents to have positively enhanced their attitude to seek healthcare in their respective HCPs. “I must tell you, ASHIA made me have the confidence to be visiting the hospital and I'm happy” (Male, Private, Rural- AEAGUF02).

### Experience of service utilization within SSHIS

The finding from the qualitative study shows that enrollment in SSHIS motivated and improved the enrollees' confidence and willingness to seek and access health care at the hospital when sick to the extent that enrollees who would not normally access care from other HCPs because of the reasons highlighted above started using the hospital as their first point of call when sick*.* An enrollee from one of the urban facilities narrated,

“The first time I came here to register, I came with the previous experience of high bills, when I finished what I spent was not up to 700 Naira, it was like a film to me [nod]. The drugs they gave to me and everything I complain that was wrong with me, I went home and within two days I took the drugs everything became okay. I tell you, it now makes me, if I feel slight headache and managed to get transportation fee I will come here because I know that all my ailment will go and too there’s not much bill to pay, I tell you that is what gladdens my heart the most” (Female, Private, Urban—NSNNEF01).

Another respondent accessing care in a rural facility said, “Yes, the experience is a good one, before I always suffer from malarial and typhoid but now, I have entered ASHIA, there’s a huge difference.….. Since I entered ASHIA and received treatment, malarial and typhoid were completely cured. I have been treated with malarial and typhoid” (Female, Public, Rural—AEUMUF01).

## Discussion

This paper examined the influence of health insurance on the care-seeking behaviour of ASHIS enrollees pre- and post-enrolment. The finding shows that there was a positive change (improvement in appropriate HSB) in enrollees' care behavoiur after their enrollment into the scheme. The improvements in the timeliness of appropriate action when ill or perceived ill-health suggests that the scheme must have achieved its objective of improving access to quality health care by reducing the financial barriers associated with poor utilization of health services. This has implications for efforts towards achieving UHC as well as SDGs Goal 3 in the study state. Our finding is consistent with previous studies in LMICs reported by SHIELD [29], Chomi et al. [17], Jutting [30], Asibey and Agyemang [31] Robyn et al. [12], and Mensah et al. [32] who found a significant association with health insurance and care-seeking behaviour. However, the finding is in contrast with another study which reported that health insurance has an effect on increased utilization of healthcare services, particularly in situations where an insured individual is still able to access quality care in the absence of insurance [33]. However, a notable concern with analysis of the influence of health insurance on care-seeking behaviour is the potential presence of a moral hazard that had been previously reported [34, 35].

The study reveals a positive and statistically significant association between positive change in HSB and some socio-demographic characteristics of the ASHIS enrollees. This implies that the HSB of the enrollees are influenced by their level of education, marital status, occupation, and the ownership of the health facility they use. People who are more educated tend to have higher knowledge of the benefits of appropriate HSB than the less educated. Moreso, private hospital seems to perform better than public facilities in term of quality of care and skilled health provider which might have contributed to the positive HSB observed in this study. Our study agrees with a previous one which stated that the extent of healthcare use is associated with socioeconomic and personal characteristics [12].

The positive significant difference found in relation to the choice of providers indicates that enrollees are more likely to choose a health facility, over other types of HCPs including herbalists, PMVs, or self-medication when offered a better and qualified facility. Affordability/cost of healthcare and good quality (availability of drugs, skilled health providers, particularly availability of doctors) were the main reasons for positive change in seeking care among SSHIS enrollees. The finding is not surprising because owing to the cost of health care, people are likely to resort to self-medication, by-pass qualified health providers [36], and substitute informal providers such as PMVs as an option of care [37]. In Nigeria, the cost of care could be attributable to one of the reasons people abandon formal health facilities for self-medication or informal providers for treatment, until the late presentation of cases which usually leads to preventable case fatalities [38]. Financial barriers including direct and indirect costs influence healthcare-seeking practices [39–41]. Undoubtedly, the scheme can be said to provide a platform for financial-risk protection among its members. Our finding is in line with previous studies that reported positive effect health insurance has on insured people using formal health providers than the uninsured in other countries [42, 43]. In contrast, another study documented minimal effect of health insurance on the HSB [44].

The perceived superior quality of care provided by the formal HCPs played a significant role in the enrollees’ choice of provider when ill. This is because when the enrollees’ expectations are not met, there is a possibility that most of them will not hesitate to seek alternatives even if it means forgoing their health insurance benefits. This has been elucidated in our results where a majority of the ASHIS enrollees who previously sought care from informal HCPs/sources are no longer patronizing the latter.

In a broader context, one of the goals of expanding or decentralizing health insurance in the Nigerian health system was to achieve UHC by 2030 by providing access to quality, affordable and efficient health care to every citizen. For a system to be universal, it has to provide a range of accessible services ‘for all’, based on needs while requiring equitable payment for the services rendered [45, 46]. Evidence shows that this can only be achieved in a social health insurance system due to its redistributive nature, enabling cross-subsidization and risk-sharing among the citizenry. In this study, our results have shown increase or improvement in care-seeking for quality services among the ASHIS enrollees without financial constraint or hardship, hence, addressing the huge health inequities and high OOPE in Anambra state.

A major strength of this study is the use of mixed methods; qualitative and quantitative (pre- and post-service utilization) which largely contributed to the robustness of the findings because it enabled complementarities and validation findings through data triangulation. Secondly, the sampling method employed allowed for diverse responses from the geographical locations, type/ownership of health facilities, and combination of respondents from the formal and informal sectors.

Our study has some limitations. First, the results of our sample may not be generalizable to other states in Nigeria, since our data collection was done in one state out of 36 states that are implementing state-based social health insurance. However, values highlighted in this study may be transferable to similar contexts. Secondly, although the enrollees' HSB improved after enrollment in the scheme, it remains unknown whether health insurance has a significant effect on their health outcomes, which this study did not estimate, thus highlighting an area for further study. Another limitation of the study is the lack of a control group, which may have implications for internal validity. However, the study was not a true experimental design. Finally, there may be a possibility of recall bias in self-reported use of healthcare, particularly before enrollment into ASHIS, however, information elicited by the tools is not too specific that a respondent would not be able to accurately recall.

## Conclusion

Our study has established that health insurance is an effective strategy for improving healthcare-seeking behaviour. It particularly highlights health insurance's influence on positive or appropriate HSB in the context of ‘when to seek care', and ‘from where to seek care’ during illness episodes or in situations requiring medical attention by preventing delays and use of informal or alternative forms of healthcare including PMVs, self-medication, traditional medicine. Thus, expanding coverage of health insurance among the uninsured is crucial in improving access to quality, affordable and efficient health care for every citizen in Anambra state.

## Data Availability

The datasets generated and analyzed in this study are not publicly available due to limitations of ethical approval involving patient data and anonymity but are available from the corresponding author on reasonable request.

## Abbreviations

ASHIA: Anambra State Health Insurance Agency
ASHIS: Anambra State Health Insurance Scheme
FGD: Focus group discussions
HCP: Health Care Providers
HSB: Health Seeking Behavoiur
OOPE: Out-of-pocket Expenditure
PMV: Patent Medicine Vendor
SMOH: State Ministry of Health
SHIS: Social Health Insurance Scheme

## References

[1] United Nations. Transforming our World: the 2030 Agenda for Sustainable Development. 2015. New York, UN; 2015 https://sustainabledevelopment.un.org/post2015/transformingourworld/publication.

[2] Levine D, Polimeni R, Ramage I (2016). Insuring health or insuring wealth? An experimental evaluation of health insurance in rural Cambodia. J Dev Econ.

[3] Cuong NV, Linh VH (2018). The impact of migration and remittances on household welfare: evidence from Vietnam. J Int Migration Integr.

[4] Olenja J (2003). Editorial Health seeking behaviour in context. East Afr Med J.

[5] Akinyemi JO, Banda P, De Wet N, Akosile AE, Odimegwu CO (2019). Household relationships and healthcare seeking behaviour for common childhood illnesses in sub-Saharan Africa: a cross-national mixed effects analysis. BMC Health Serv Res.

[6] Latunji OO, Akinyemi OO (2018). Factors influencing health-seeking behaviour among civil servants in Ibadan. Nigeria Annals Ibadan Postgraduate Med.

[7] Mwase I. Social capital and household health-seeking behaviour for children in the context of urban neighbourhoods: The case of Khayelitsha in Western Cape, South Africa. [Thesis]. University of Cape Town, Faculty of Health Sciences, Department of Public Health and Family Medicine, 2015 [cited 2022 Apr 11]. Available from: http://hdl.handle.net/11427/13806

[8] MacKian S (2003). A review of health seeking behaviour: problems and prospects Health Systems. Development Programme.

[9] Kamaruzaman NA, Muthupalaniappen L, Pasi H (2013). Health care seeking behavior among caregivers of children with pneumonia in a rural area. Int Med J.

[10] Buor D. Determinants of utilization of health services by women in rural and urban areas in Ghana. International J Human Geography Environ Sci. 2004;61(1):89–102.

[11] Saeed BI, Oduro SD, Ebenezer AMFE, Zhao X (2012). Determinants of healthcare utilization among the ageing population in Ghana. Int J Bus Soc Sci.

[12] Robyn PJ, Hill A, Liu Y, Souares A, Savadogo G, Sié A, Sauerborn R (2011). Econometric analysis to evaluate the effect of community-based health insurance on reducing informal self-care in Burkina Faso. Health Policy Plann.

[13] Goudge J, Alaba OA, Govender V. et al. Social health insurance contributes to universal coverage in South Africa, but generates inequities: survey among members of a government employee insurance scheme. Int J Equity Health 2018; 17, 1. 10.1186/s12939-017-0710-z 10.1186/s12939-017-0710-z PMC5755208 29301537

[14] Ataguba J, Akazili J (2010). Health care financing in South Africa: moving towards universal coverage. Continuing Med Educ.

[15] Anderson M, Dobkin C, Gross T (2012). The effect of health insurance coverage on the use of medical services”. Am Econ J Econ Pol.

[16] Gustafsson-Wright E, Janssens W, van der Gaag J (2011). The inequitable impact of health shocks on the uninsured in Namibia. Health Policy Plan.

[17] Chomi, E. N., Mujinja, P. G.M., Enemark, U., Hansen, K., & Kiwara, A. D. (2014). Health care seeking behavior and utilization in a multiple health insurance system: does insurance affiliation matter. International J Equity Health. 13(25). 10.1186/1475-9276-13-25. 10.1186/1475-9276-13-25 PMC3994926 24645876

[18] Smith P, Witter S (2004). Risk pooling in health care financing: the implications for health system performance. HNP Discussion Paper.

[19] Ekman B (2007). The impact of health insurance on outpatient utilisation and expenditure: evidence from one middle-income country using national household survey data. Health Res Policy Syst.

[20] Towards Universal Health Coverage in Nigeria: The States at the driving Seat (2015). Report of work by the Technical Working Group (TWG) to develop a Template to guide States set up their Health Insurance Schemes.

[21] Anambra State Government (2016). Anambra State Health Insurance scheme Law.

[22] State Ministry of Health. Anambra State Health Insurance Operational Guideline and Benefits Package. State Ministry of health, Awka, Anambra State, Nigeria; 2019. https://ashia.an.gov.ng/

[23] State Ministry of Health (2019). Anambra State Health Financing Policy & Strategy.

[24] State Ministry of Health (2018). Anambra State Strategic Health development Plan 2018–2022: ensuring healthy lives and promoting the wellbeing of Nigerian populace at all ages.

[25] National Population Commission (NPC) [Nigeria] and ICF (2019). Nigeria Demographic and Health Survey 2018.

[26] Ogunlesi TA, Olanrewaju DM (2010). Sociodemographic factors and appropriate health careseeking behavior for childhood illnesses. J Trop Pediatr.

[27] Anderson R (1968). A behavioral theory of families’ use of health services.

[28] Andersen RM (1995). Revisiting the behavioral model and access to medical care: does it matter?. J Health Soc Behav.

[29] SHIELD (2011). Who is covered by health insurance schemes and which services are used in Tanzania? Policy Brief. Shield Health Financing Reform.

[30] Jutting JP (2004). Do community-based health insurance schemes improve poor people’s access to health care? Evidence from rural Senegal. World Dev.

[31] Asibe and a Benedict Osei Asibey and Seth Agyemang Analysing the Influence of Health Insurance Status on Peoples’ Health Seeking Behaviour in Rural Ghana. J Tropical Med. 2017;1–7 10.1155/2017/8486451 10.1155/2017/8486451 PMC5439069 28567060

[32] MENsa Mensah J, Oppong JR, Bobi-Barimah K, Frempong G, Sabi W: An evaluation of the Ghana National Health Insurance Scheme in the context of the health MDGs. Global Development Network Working Paper Series 2010, No. 40. GDN.

[33] Raza WA, Van de Poel E, Bedi A, Rutten F (2016). Impact of Communitybased Health Insurance on Access and Financial Protection: evidence from Three Randomized Control Trials in Rural India. Health Econ.

[34] Boone J (2015). Basic versus supplementary health insurance: moral hazard and adverse selection. J Public Econ.

[35] Haddad GK, Anbaji MZ (2010). Analysis of adverse selection and moral hazard in the health insurance market of Iran. Geneva Papers Risk Insurance-Issues Pract.

[36] Gotsadze G, Bennett S, Ranson K, Gzirishvili D (2005). Health careseeking behaviour and out-of-pocket payments in Tbilisi. Georgia Health Policy Plan.

[37] Deolalikar A (2002). Access to health services by the poor and the nonpoor: The case of Vietnam. J Asian Afr Stud.

[38] Abdulraheem IS, Parakoyi DB (2009). Factors affecting mothers’ healthcareseeking behavior for childhood illnesses in a rural Nigerian Setting. Early Child Dev Care.

[39] Odu BP, Mitchell S, Isa H, Ugot I, Yusuf R, Cockcroft A (2015). Equity and seeking treatment for young children with fever in Nigeria: a cross-sectional study in Cross River and Bauchi states. Infect Dis Poverty.

[40] Colvin CJ, Smith HJ, Swartz A, Ahs JW, de Heer J, Opiyo N (2013). Understanding careseeking for child illness in sub-Saharan Africa: a systematic review and conceptual framework based on qualitative research of household recognition and response to child diarrhoea, pneumonia and malaria. Soc Sci Med.

[41] Awoyemi TT, Obayelu OA, Opaluwa HI (2011). Effect of distance on utilization of health care services in rural Kogi state. Nigeria J Hum Ecol.

[42] Chankova S, Sulzbach S, Diop F (2008). Impact of mutual health organizations: Evidence from West Africa. Health Policy Plan.

[43] Yip W, Wang H, Hsiao W (2007). The impact of rural mutual health care on access to care: Evaluation of a social experiment in rural China.

[44] Jutting J, Tine J. Micro insurance schemes and health care provision in developing countries: An empirical analysis of the impact of mutual health insurance schemes in rural Senegal. Center for development research (ZEF) Bonn ILO/ZEF-Project No. 7359 Project report: 5. Germany: ZEF; 2000.

[45] EQUINET. Challenging inequity through redistributive health systems. In Regional Equity Watch 2012: Assessing progress towards equity in health in East and Southern Africa. Harare: EQUINET; 2012.

[46] McIntyre D. Health service financing for universal coverage in east and southern Africa. In EQUINET Discussion Paper. No. 95. Health Economics Unit (UCT), Harare: EQUINET; 2012

